# LIMITED DIAGNOSTIC ACCURACY OF FIB-4 FOR DETECTING SIGNIFICANT LIVER FIBROSIS IN CHRONIC HEPATITIS B: A CROSS-SECTIONAL STUDY

**DOI:** 10.1590/S0004-2803.24612025-085

**Published:** 2026-07-20

**Authors:** Elyas Abdullah ALSALAMY, Heba Mahmoud Taha ELWESHAHI, Heba Hany OMAR, Ahmed KAMAL

**Affiliations:** 1 Faculty of Medicine, Alexandria University-Manchester University, Joint MBChB Programme, Alexandria, Egypt.; 2 Faculty of Medicine, Alexandria University, Professor of Public Health, Alexandria, Egypt.; 3 Alexandria University, University Hospitals of Alexandria, Fellow of Microbiology and Head of Pulmonology Clinical Pharmacy, Alexandria, Egypt.; 4 Faculty of Medicine, Alexandria University, Assistant Professor of Internal Medicine and Hepatology, Alexandria, Egypt.

**Keywords:** FIB-4, hepatitis B, liver fibrosis, vibration controlled transient elastography, FIB-4, hepatite B, fibrose hepática, elastografia transitória controlada por vibração

## Abstract

**Background:**

Liver disease linked to the hepatitis B virus (HBV) is a serious public threat worldwide and in Egypt. The incidence of HBV infection in Egypt is about 1.4%. Accurate liver fibrosis assessments are crucial in the evaluation and follow-up of HBV patients, but traditional biopsies have limitations, prompting the use of non-invasive methods like the vibration controlled transient elastography (VCTE), which may not be available in limited-resource treatment centers.

**Objective:**

This study aimed to assess the diagnostic accuracy of Fibrosis-4 (FIB-4) and Aminotransferase to Platelet Ratio Index (APRI) compared to VCTE in detecting significant liver fibrosis in adult chronic HBV patients, potentially offering a more accessible and less costly approach to disease management. **Methods** - In this cross-sectional study, 247 subjects with chronic HBV from Alexandria University Viral Hepatitis Treatment Unit were included. Data collected included demographic details, HBV viral load, liver function tests, Complete Blood Count (CBC), international normalized ratio (INR), prothrombin time, albumin, total and direct bilirubin, APRI score, FIB-4 score, imaging, and VCTE data. IBM SPSS software was used for statistical analysis, with qualitative and quantitative data described appropriately. Logistic regression and receiver operating characteristic curve (ROC) curves were used to identify significant predictors of advanced fibrosis and assess diagnostic performance.

**Results:**

Based on VCTE results, 247 chronic hepatitis B patients were classified into two groups: 140 with no or mild fibrosis (F0/1) and 107 with significant fibrosis (F2-F4). Significant differences were found in hemoglobin, albumin, and INR levels between the groups. The FIB-4 and APRI scores were significantly higher in the significant fibrosis group, with median FIB-4 scores of 1.26 vs 0.79 and median APRI scores of 0.40 vs 0.27. The FIB-4 index remained a significant predictor of significant fibrosis in multivariate analysis, with an odds ratio of 1.558. At a cut-off value of >0.88, the FIB-4 score achieved a sensitivity of 65.42% and a specificity of 60.0% in differentiating patients with and without significant fibrosis.

**Conclusion:**

Although the use of FIB-4 score is potentially useful in resource-limited settings, a significant number of misclassifications can occur due to its moderate sensitivity and specificity which necessitate further incorporation of additional diagnostic methods to ensure the accurate staging of liver fibrosis.

## HIGHLIGHTS

• Distinguishing the severity of liver fibrosis stage is clinically important in prescribing antiviral therapies, and in follow up of the patients.

• FIB-4 index is a valuable and readily available tool for the non-invasive assessment of liver fibrosis in HBV patients but of only fair diagnostic accuracy.

• Future research studies incorporating other tests like ELF score, novel biomarkers, machine learning &/or artificial intelligence imaging models to improve the sensitivity and specificity of FIB-4 score is needed.

## RESUMO

**Contexto:**

A doença hepática associada ao vírus da hepatite B (VHB) representa uma séria ameaça para a saúde pública mundial e também no Egipto. A incidência de infecção pelo VHB no Egipto é de aproximadamente 1,4%. Avaliações precisas da fibrose hepática são cruciais na avaliação e no seguimento dos doentes com VHB, no entanto as biópsias tradicionais apresentam limitações, o que justifica a utilização de métodos não invasivos, como a elastografia transitória controlada por vibração (ETCV), que pode não estar disponível em centros de tratamento com recursos limitados.

**Objetivo:**

Este estudo teve como objetivo avaliar a precisão diagnóstica do Índice de Fibrose-4 (FIB-4) e do Índice de Razão Aminotransferase/Plaquetas (APRI) em comparação com a ETCV na detecção de fibrose hepática significativa em doentes adultos com VHB crónico, oferecendo potencialmente uma abordagem mais acessível e menos dispendiosa para a gestão da doença.

**Métodos:**

Neste estudo transversal foram incluídos 247 indivíduos com VHB crónico da Unidade de Tratamento de Hepatite Viral da Universidade de Alexandria. Os dados recolhidos incluíram informação demográfica, carga viral do VHB, testes de função hepática, hemograma completo (HC), razão normalizada internacional (RNI), tempo de protrombina, albumina, bilirrubina total e direta, pontuação APRI, pontuação FIB-4, exames de imagem e dados de elastografia transitória controlada por vídeo (ETCV). O software IBM SPSS foi utilizado para a análise estatística, tendo os dados qualitativos e quantitativos sido adequadamente descritos. A regressão logística e as curvas ROC (características de operação do recetor) foram utilizadas para identificar preditores significativos de fibrose avançada e avaliar o desempenho diagnóstico.

**Resultados:**

Com base nos resultados da ETCV, 247 doentes com hepatite B crónica foram classificados em dois grupos: 140 com ausência ou fibrose ligeira (F0/1) e 107 com fibrose significativa (F2-F4). Foram encontradas diferenças significativas nos níveis de hemoglobina, albumina e RNI entre os grupos. Os scores FIB-4 e APRI foram significativamente mais elevados no grupo com fibrose significativa, com uma mediana de score FIB-4 de 1,26 vs 0,79 e uma mediana de score APRI de 0,40 vs 0,27. O índice FIB-4 manteve-se um preditor significativo de fibrose significativa na análise multivariada, com uma razão de probabilidades de 1,558. Com um valor de corte >0,88, o score FIB-4 atingiu uma sensibilidade de 65,42% e uma especificidade de 60,0% na diferenciação entre doentes com e sem fibrose significativa.

**Conclusão:**

Embora a utilização do score FIB-4 seja potencialmente útil em ambientes com recursos limitados, um número significativo de classificações incorretas pode ocorrer devido à sua sensibilidade e especificidade moderadas, o que exige a incorporação de métodos de diagnóstico adicionais para garantir o estadiamento preciso da fibrose hepática.

## INTRODUCION

Hepatitis B virus (HBV) associated liver disease is the most important etiology of cirrhosis worldwide. Significant liver inflammation and scarring are the driving forces for hepatic decompensation and development of hepatocellular carcinoma^1^. On a global scale, a third of people are thought to have been exposed to HBV. Egypt has one of the highest incidence rates of HBV (1.6%) in the Eastern Mediterranean Region, making it a significant public health concern^2^.

Accurate staging of liver fibrosis stage is crucial in figuring out the need for antiviral therapies, and in follow up of the patients^3^ ^-^ ^5^. The recent WHO guidelines recommend treating chronic hepatitis B patients with significant fibrosis whatever the viremia level, while the last EASL guidelines recommend treating patients with advanced fibrosis with any detectable level of HBV DNA^3^ ^-^ ^4^. Conventional liver biopsy is associated with certain limitations, including possible morbidity, inter- and intra-observer variability, sampling errors, patients’ reluctance, and high costs for the evaluation of tissue specimens^6^. So, non-invasive evaluation of liver fibrosis in patients with HBV infection is of particular importance as it can help in the management decisions and follow up of the patients without the need of liver biopsy^7^. Vibration controlled transient elastography (VCTE) has gained popularity in assessing liver fibrosis stage among HBV-infected patients, but it is not available in all centers managing HBV, needs special experience, and has some limitations like in obese patients^8^. This brought WHO and EASL to recommend using laboratory based non-invasive tools, namely APRI and FIB-4 for the assessment of liver fibrosis stage^3^ ^,^ ^4^. These formulas are well established in patients with chronic hepatitis C, while their use in patients with chronic hepatitis B is still questionable^9^. In this study, we investigated the accuracy of Fibrosis-4 (FIB-4), and Aminotransferase to Platelet Ratio Index (APRI) scores, in comparison to VCTE for determining the stage of fibrosis in adults chronically infected with HBV.

## METHODS

In this cross-sectional study, 247 patients from the Alexandria University Viral Hepatitis Treatment Unit who had a chronic HBV infection were included. The study was carried out between January 15, 2024, and May 15, 2024.

The minimum sample size was estimated using MedCalc software using data reported by Rungta et al. 2021, who showed expected area under the curve (AUC) for FIB-4 for diagnosing significant fibrosis of 0.753. The minimum sample size at 5% level of significance to achieve 80% power was 80^10^.

Patients with combined hepatitis C Virus (HCV) infection &/or Human Immunodeficiency Virus (HIV) infection, and patients with blood malignancies were excluded. Also, patients with decompensated liver cirrhosis were not included in the study.

Patient data were extracted from medical records of the patients, ensuring a robust and reliable data set. The patient information collected included age and gender. In addition to demographic details, relevant data was also procured. This included the viral load of HBV in the patients, liver function tests, Complete Blood Count (CBC), international normalized ratio (INR) and the prothrombin time. Added to those data, the level of Aminotransferase to Platelet Ratio Index (APRI) score and the Fibrosis-4 (FIB-4) score were also obtained^7^.

Regarding imaging, ultrasound liver, spleen, and portal vein assessment were recorded. VCTE results were also reported. Measures at or above 7.25 kPa was considered as significant fibrosis (F2 or more)^11^, measures at 9.3 kPa or above were considered as advanced fibrosis (F3 or more)^12^, while measures at 12.4 kPa were considered as cirrhosis (F4)^11^.

An ethical approval was obtained from the ethical committee at Faculty of medicine, Alexandria University with serial number 0306473, IRB number: 00012098.

### Statistical analysis

The data was examined using the IBM SPSS software package version 20.0 (IBM Corp., Armonk, NY). The qualitative data was described using numbers and percentages. The Shapiro-Wilk test was used to determine if the distribution of the quantitative data was normal or not. Quantitative data were described using either the median and interquartile range (IQR) or the mean and standard deviation. The significance of the findings was evaluated using the 5% criterion of significance.

Qualitative data between groups were compared using the chi-square test or Fisher’s exact test (chi-square adjustment when more than 20% of cells had an expected count below 5). Quantitative data were subjected to the Mann Whitney or t test.

A receiver operating characteristic curve (ROC) was produced for both FIB-4 and APRI by plotting sensitivity (TP) on the Y axis against 1-specificity (FP) on the X axis at different cutoff points. The Youden index was used to establish the cut-off. The diagnostic performance is indicated by the area under the ROC curve.

### Ethics statements

Ethical approval was obtained from the ethical committee at Faculty of medicine, Alexandria University with serial number: 0306473 IRB: 00012098. An informed consent was obtained from all subjects included in the study.

## RESULTS

Based on the results of VCTE, the studied patients were classified into five stages of hepatic fibrosis (from F0 to F4). The patients were bifurcated into two distinct groups; the first group consisted of 140 patients, with no or mild fibrosis (F0-F1) with VCTE measurement <7.25 kPa. The second group consisted of 107 patients with significant fibrosis (F2-F4) with VCTE measurement ≥7.25 kPa. Of them 38 were F2, and 14 were F3, while the remaining 55 patients were F4. The group with significant fibrosis had a considerably higher age (Table 1).

**TABLE 1:** Distribution of the cases under study according to the age and gender.

	Total (n=247)		No /mild fibrosis		Significant fibrosis		Test of Significance	***P*-value**
			F0-F1 (n=140)		F2-F4 (n=107)			
	No.	%	No.	%	No.	%		
Gender								
Male	122	49.4	70	50.0	52	48.6	χ^2^=0.048	0.827
Female	125	50.6	70	50.0	55	51.4		
Age (years)								
Min. - Max.	18.0-82.0		18.0-78.0		20.0-82.0		t=3.777*	<0.001*
Mean ± SD.	44.61±12.55		42.04±12.09		47.97±12.41			

IQR: inter quartile range. SD: standard deviation. t: student t-test. c^2^: chi square test. Min: minimum. Max: maximum. *: statistically significant at *P*≤0.05.

The key findings in Table 2 reveal significant differences between patients with and without significant fibrosis in several parameters: (Table 2).

**TABLE 2:** Comparison of the two groups under study based on laboratory investigations.

	Total	No /mild fibrosis	Significant fibrosis	U	***P*-value**
	(n=247)	F0 - F1 (n=140)	F2 - F3 - F4 (n=107)		
**Hb**	13.0	13.0	13.0	6300.50^*^	0.032^*^
Median (IQR)	(11.75-14.50)	(12.0-15.0)	(11.0-14.20)		
**Albumin**	4.30	4.30	4.10	6099.0^*^	0.012^*^
Median (IQR)	(3.90-4.60)	(4.0-4.60)	(3.75-4.50)		
**INR**	1.01	1.0	0.07	5792.0^*^	0.002^*^
Median (IQR)	(1.0-1.11)	(0.98-1.10)	(1.0-1.19)		
**WBC**	6.50	6.58	6.50	6990.50	0.369
Median (IQR)	(5.0-7.85)	(5.0-7.96)	(4.97-7.58)		
**PLT**	226.0	237.0	191.0	5413.50^*^	<0.001^*^
Median (IQR)	(168.5-281.5)	(190.5-287.5)	(130.0-262.0)		
**Neutrophils**	3.40	3.55	3.20	6782.50	0.203
Median (IQR)	(2.50-4.32)	(2.50-4.58)	(2.55-4.0)		
**Lymphocytes**	2.04	2.10	2.00	7216.50	0.623
Median (IQR)	(1.50-2.60)	(1.60 - 2.50)	(1.38-2.70)		
**ALT**	32.0	31.0	32.0	6763.0	0.191
Median (IQR)	(24.0-48.0)	(23.0-45.50)	(19.50-35.0)		
**AST**	26.0	24.0	31.0	5944.0^*^	0.005^*^
Median (IQR)	(21.0-37.0)	(26.0-50.0)	(22.50-41.0)		
**TSB**	0.50	0.50	0.50	7297.0	0.726
Median (IQR)	(0.40-0.70)	(0.30-0.70)	(0.40-0.70)		
**INR**	1.01	1.0	1.07	5792.0^*^	0.002^*^
Median (IQR)	(1.0-1.11)	(0.98-1.10)	(1.0-1.19)		
**Creatinine**	0.70	0.70	0.70	7248.50	0.661
Median (IQR)	(0.60-0.90)	(0.60-0.90)	(0.60-0.90)		
**NLR**	1.58	1.59	1.50	7248.0	0.664
Median (IQR)	(1.18-2.22)	(1.18-2.23)	(1.18-2.18)		
FIB-4	0.92	0.79	1.26	4675.50^*^	<0.001^*^
Median (IQR)	(0.61-1.53)	(0.55-1.11)	(0.75-2.39)		
**APRI score**	0.33	0.27FIB-4	0.40	5097.0^*^	<0.001^*^
Median (IQR)	(0.20-0.52)	(0.18-0.41)	(0.23-0.87)		

IQR: inter quartile range. SD: standard deviation. U: mann whitney test. *: statistically significant at *P*≤0.05. WBC: white blood count. ALT: alanine aminotransferases. AST: aspartate aminotransferases. INR: international normalized ratio. NLR: neutrophil to lymphocyte ratio. TSB: total serum bilirubin. FIB-4: fibrosis-4. APRI: aminotransferase to platelet ratio index.

Hemoglobin: the significant fibrosis group had lower mean hemoglobin levels (12.49±2.28 g/dL) than the other group (13.14±2.09 g/dL) (*P*=0.032).

Albumin: the significant fibrosis group had a considerably lower mean Albumin level (4.11±0.55 g/dL against 4.29±0.47 g/dL) (*P*=0.012).

International Normalized Ratio (INR): the significant fibrosis group had a significantly higher mean INR (1.11±0.23 vs 1.04±0.14) (*P*=0.002).

There were significant differences in FIB-4 and APRI scores between the two patient groups. The median FIB-4 score was 0.79 (IQR: 0.55 - 1.11) in the F0 - F1 group and 1.26 (IQR: 0.75 - 2.39) in the significant fibrosis group (U=4675.50, *P*<0.001). Likewise, the APRI scores were significantly different across the two groups, with a median score of 0.27 (IQR: 0.18 - 0.41) in the F0 - F1 group compared to 0.40 (IQR: 0.23 - 0.87) in the F2 - F4 group (U=5097.0, *P*<0.001). In contrast, no considerable difference was detected in the Neutrophil to Lymphocyte Ratio (NLR) between the two groups (U=7248.0, *P*=0.664).

In Table 3, the univariate analysis revealed that lower hemoglobin, lower albumin, higher INR, and higher FIB-4 scores exhibited a significant relationship with the presence of significant fibrosis. In the multivariate analysis, the FIB-4 index remained significant with a *P*-value of 0.001. The odds ratio was at 1.558, meaning that for each unit increase in FIB-4, the odds of the presence of significant fibrosis increased by approximately 56%, within a 95% confidence interval of 1.203 to 2.017. The other variables, however, did not maintain their significance in this model.

**TABLE 3:** Logistic regression analysis, both univariate and multivariate, for the predictors of significant fibrosis.

	Univariate		^#^ **Multivariate Model-3**	
	***P*-value**	OR (LL - UL 95%CI)	***P*-value**	OR (LL - UL 95%CI)
**Hb**	0.024^*^	0.873 (0.776-0.982)	0.985	0.999 (0.870-1.147)
**Albumin**	0.008^*^	0.504 (0.303-0.836)	0.201	0.685 (0.384-1.224)
**INR**	0.003^*^	21.571 (2.747-169.365)	0.164	3.962 (0.571-27.493)
**FIB-4**	<0.001^*^	1.713 (1.329-2.207)	0.001^*^	1.558 (1.203-2.017)

LL: lower limit. UL: upper limit. CI: confidence interval. INR: international normalization ratio.

The FIB-4 score achieved an Area under the Curve (AUC) of 0.686 (*P*<0.001, 95% Confidence Interval (CI): 0.619 - 0.753), demonstrating a fair diagnostic accuracy in distinguishing HBV patients with significant fibrosis. At a cut-off value of >0.88, the FIB-4 score exhibited a sensitivity of 65.42% and a specificity of 60.0%, with Positive Predictive Value (PPV) and Negative Predictive Value (NPV) of 55.6% and 69.4% respectively.

**FIGURE 1:**
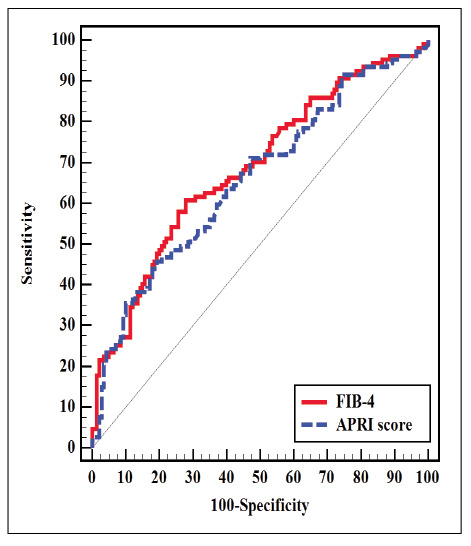
ROC curve for FIB-4 and APRI scores to discriminate patients with significant fibrosis among patients with chronic hepatitis B virus infection.

Similarly, the APRI score showed an AUC of 0.660 (*P*<0.001, 95%CI: 0.591 - 0.729), indicating a fair diagnostic performance in distinguishing HBV patients with significant fibrosis. With a cut-off value of >0.32, the APRI score had a sensitivity of 63.55% and a specificity of 60.0%, along with a PPV and NPV of 54.8% and 68.3% respectively (Table 4 and Figure 1).

**TABLE 4:** Diagnostic performance for FIB-4 and APRI score to discriminate Significant fibrosis (F2 - F4) (n=107) from patients without significant fibrosis (F0 - F1) (n=140).

	AUC	***P*-value**	95%CI	Cut off	Sensitivity	Specificity	PPV	NPV
FIB-4	0.686	<0.001^*^	0.619-0.753	>0.88766	65.42	60.0	55.6	69.4
APRI score	0.660	<0.001^*^	0.591-0.729	>0.32480	63.55	60.0	54.8	68.3

AUC: area under the curve. CI: confidence interval. PPV: positive predictive value. NPV: negative predictive value. FIB-4: fibrosis-4. APRI: aminotransferase to platelet ratio index.

## DISCUSSION

Differentiating between HBV-infected patients with and without significant fibrosis is important in management plan selection and in the patient’s follow up^3^ ^-^ ^5^. The gold standard procedure, liver biopsy, is an invasive one, while VCTE needs special expertise, an expensive machine and not available in many centers^6^ ^-^ ^8^. So, it is crucial to assess the accuracy of the available non-invasive tools as possible substitute methods to ascertain whether these patients have significant fibrosis.

In our study, hemoglobin showed a significant *p*-value of 0.024 in the univariate model, with an odds ratio (OR) of 0.873. This suggests a potential inverse relationship between hemoglobin levels and the degree of fibrosis. However, in the multivariate model, the *P*-value was 0.985, which is not statistically significant. Albumin demonstrated a significant *P*-value of 0.008 in the univariate model, with an OR of 0.504. But again, in the multivariate model, albumin was insignificant with a *P*-value of 0.201. For INR, we found a significant *P*-value of 0.003 in the univariate model, with an OR of 21.571. However, in the multivariate model, the *P*-value was 0.164, which is not statistically significant.

Both FIB-4 and APRI scores have gained popularity in liver fibrosis assessment. In our study, FIB-4 was significant in both the univariate (*P*<0.001, OR=1.713) and multivariate (*P*=0.001, OR=1.558) models. This result suggests that the FIB-4 index could serve as a robust and reliable tool in routine clinical practice. But an AUC of 0.68 indicates only fair diagnostic accuracy with suboptimal sensitivity and specificity at the cut-off point of 0.88. A meta-analysis on the diagnostic value of FIB-4 in the assessment of liver fibrosis among HBV patients showed that the diagnostic accuracy was not very high with heterogeneity between studies regarding the cut-off values^13^. SONIC-B study even suggested 0.7 as a cut-off point for FIB-4 to avoid misclassifying patients with cirrhosis, not only significant fibrosis^10^. Itakura et al. (2021)^14^ concluded that the use of FIB-4 and APRI can act as practical tools to evaluate the grading of liver fibrosis among HCV but not HBV patients. In our study APRI score accuracy in determining patients with significant fibrosis was not optimal. So, while FIB-4 and APRI offer affordable and accessible options, their diagnostic accuracy remains modest, limiting standalone clinical use.

Gür-Altunay and Yürük-Atasoy (2023)^15^ evaluated APRI and FIB-4 in patients chronically infected with HBV for liver fibrosis assessment against liver biopsies results. They concluded that while APRI and FIB-4 provide insight into fibrosis status, liver biopsies are still needed for accurate evaluation. On the other hand, VCTE was shown to have high accuracy in determining fibrosis stages in HBV patients^16^. This raises many concerns on how to adhere to the new HBV management guidelines in limited resources centers. This directly opens the door for research into combining existing tools or identifying new ones. Combining markers that reflect different aspects of liver injury, extracellular matrix remodeling, and fibrosis could provide a more accurate tool for fibrosis stage assessment. For example, further incorporation of other tests like ELF score, other future novel biomarkers, machine learning &/or artificial intelligence (AI) imaging models may be beneficial but needs more studies^17^ ^-^ ^19^.

Our study has limitations due to its retrospective nature and single-center design. However, our findings could pave the way for multicenter prospective studies that aim to evaluate FIB-4 and APRI scores in chronic hepatitis B patients, with the goal of refining the current guidelines recommendations. Since VCTE is currently a validated tool with high accuracy and is advised by the recent guidelines, we have chosen it as the reference method in this study in light of the current recommendation against the routine use of liver biopsy for fibrosis degree assessment among patients with chronic hepatitis B.3^16^.

## CONCLUSION

In conclusion, this study contributes to the evidence supporting that while the use of FIB-4 score is potentially useful in resource-limited settings, a significant number of misclassifications which could impact clinical decisions can occur due to its moderate sensitivity and specificity which necessitate further incorporation of additional diagnostic methods to ensure the accurate staging of liver fibrosis.

## Data Availability

Data-available-upon-request
